# Consumption displacement in households with noncommunicable diseases in Bangladesh

**DOI:** 10.1371/journal.pone.0208504

**Published:** 2018-12-13

**Authors:** Biplab Kumar Datta, Muhammad Jami Husain, Sohani Fatehin, Deliana Kostova

**Affiliations:** 1 Global Noncommunicable Diseases Branch, Division of Global Health Protection, Center for Global Health, Centers for Disease Control and Prevention, Atlanta, GA, United States of America; 2 Department of Economics, Dickinson College, Carlisle, PA, United States of America; RTI International, UNITED STATES

## Abstract

The economic burden of noncommunicable diseases (NCDs), including treatment costs and income and productivity losses, is a growing concern in developing countries, where NCD medical expenditure may offset consumption of other essential commodities. This study examines the role of NCDs in household resource allocation in Bangladesh. We use the Bangladesh Household Income and Expenditure Survey (HIES) 2010 to obtain expenditure data on 11 household expenditure categories and 12 food expenditure sub-categories for 12,240 households. Household NCD status was determined through self-report of at least one of the six major NCDs within the household–heart disease, hypertension, diabetes, kidney diseases, asthma, and cancer. We estimated unadjusted and regression-adjusted differences in household expenditure shares between NCD and non-NCD households. We further investigated how consumption of different food sub-categories is related to NCD status, distinguishing between household economic levels. The medical expenditure share was estimated to be 59% higher for NCD households than non-NCD households, and NCD households had lower expenditure shares on food, clothing, hygiene, and energy. Regression results indicated that presence of NCDs was associated with lower relative expenditure on clothing and housing in all economic subgroups, and with lower expenditure on food among marginally poor households. Having an NCD was significantly associated with higher household spending on tobacco and higher-calorie foods such as sugar, beverages, meat, dairy, and fruit, and with lower spending on fish, vegetables, and legumes. The findings indicate a link between NCDs and the possibility of adverse economic effects on the household by highlighting the potential displacement effect on household consumption that might occur through higher medical expenditure and lower spending on essentials. The findings might also point to a need for raising awareness about the link between NCDs and diet in Bangladesh.

## Introduction

Noncommunicable diseases (NCDs) are the leading cause of death worldwide, accounting for 68% of total deaths in 2012. Among all NCD deaths, 42% are premature (before age 70), with the majority of premature deaths (86%) occurring in low-and-middle-income countries (LMICs) [1]. Premature NCD morbidity and mortality reduces labor productivity, and associated treatment costs can increase health care expenditure and erode savings, adversely affecting economic growth and development [2,3]. The adverse economic impact of NCDs is a growing concern for LMICs [4], where chronic illness can result in substantial out-of-pocket expenditure compared to other diseases [5], reduce consumption of essential goods, and increase vulnerability to various shocks [6]. However, most studies investigating NCD-associated productivity losses evaluate evidence from high-income countries [7], and less is known about the social, financial, and economic effects of NCDs in LMICs [8]. A number of studies have demonstrated significant links between NCDs and out-of-pocket health expenditure in LMICs [9,10,11,12]; rising out-of-pocket expenditure has in turn been associated with reduced clothing and education expenditure among poor households in India [6] and with reduced food expenditure in Sri Lanka [13]. Nonetheless, evidence on the consumption displacement associated with NCDs in LMICs is relatively limited.

This paper investigates the role of NCDs in household resource allocation in Bangladesh. Bangladesh is a lower-middle-income country with a large population and a rapidly growing NCD prevalence. NCD mortality in Bangladesh increased from 8% in 1986 to 68% in 2006 [14], and NCDs presently account for nearly 60% of the total disease burden [1]. As in most LMICs with low health insurance coverage, in Bangladesh the major share (63.3%) of healthcare spending occurs out-of-pocket, directly impacting household budgets [15]. NCDs have been linked to poverty and inequality in Bangladesh [16,17], where it has been estimated that larger out-of-pocket expenditure due to NCDs could push an additional 4.6% of households into poverty annually [18]. However, no prior studies have evaluated the consumption displacement effects of NCDs. Investigating the resource allocation effects of NCDs is relevant for health policy development in Bangladesh, and evidence regarding the tradeoffs faced by NCD-affected households is key for articulating appropriate NCD prevention and mitigation strategies.

## Data and methods

We analyzed household-level NCD status and monthly expenditure data from the Bangladesh Household Income and Expenditure Survey (HIES) 2010. HIES is a nationally- representative survey of 12,240 households from 16 strata comprised of rural, urban, and statistical metropolitan areas (SMA) [19]. A household was defined as NCD-affected if at least one of its members was reported to have one or more of the following NCDs within the past 12 months–heart disease, hypertension, diabetes, kidney diseases, asthma, or cancer.

HIES (2010) collects self-reported household-level data on daily, weekly, monthly, and annual expenditure, which were used to calculate average monthly consumption expenditure indicators. We classified household expenditure into 11 categories: medical, food, tobacco, clothing, housing, education, lifestyle and hygiene, energy, transportation and communication, entertainment, and miscellaneous. All categories were represented as shares of total expenditure. We further created 12 sub-categories for monthly food expenditure: cereal, pulses (legumes), fish, egg, meat, milk, sugar, oil, fruits, vegetables, beverages, and miscellaneous. All food consumption categories were represented as shares of total food expenditure.

**Fig 1:**
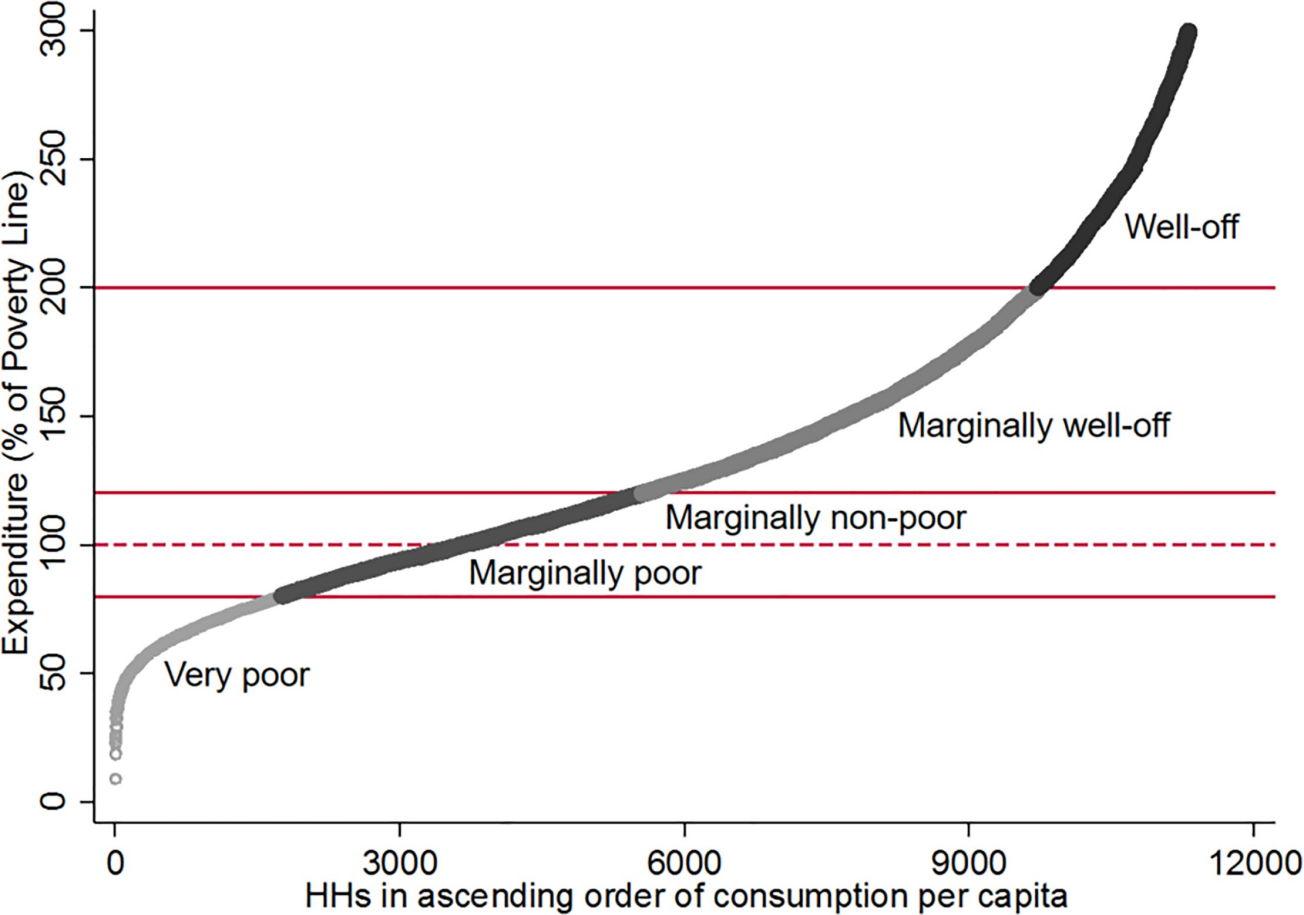
Classification of household categories. Notes: Households below poverty line but above 80% of the poverty line are considered marginally poor, and households above poverty line but below 120% of the poverty line are considered marginally non-poor. Together these two groups are categorized as marginally poor in the analysis. Poverty lines range from BDT 1,311 to BDT 2,038 [19].

To evaluate consumption effects by income level, we constructed four household income categories based on household consumption per capita relative to the stratum specific consumption poverty line [19]: very poor (<80% of the poverty line), marginally poor (> = 80% to <120% of the poverty line), marginally well-off (> = 120% to <200% of the poverty line), and well-off (> = 200% of the poverty line) (Fig 1). Descriptive statistics indicate that self-reported NCD prevalence is lowest for the very poor households and highest for the most affluent households (Table 1), suggesting the presence of an income gradient in NCD diagnosis. Across all income groups, households are more likely to report an NCD if they are larger or have elderly members.

**Table 1:** Descriptive statistics.

	All ^a^	Very Poor ^c^	Marginally Poor	Marginally Well-off	Well-off
**Proportion of NCD** ^b^ **households** (%)	22.66 (21.53,23.79)	16.11 (14.06,18.16)	18.68 (17.11,20.25)	23.42 (21.86,24.98)	32.29 (29.98,34.61)
**Average** ^d^ **Income per capita** (BDT)					
*NCD*	3080.8	1082.8	1583.8	2469.9	5934.4
	(2860.8,3300.7)	(1001.0,1164.5)	(1489.8,1677.8)	(2328.0,2611.9)	(5375.3,6493.5)
*Non-NCD*	2582.9	1199.8	1669.2	2624.4	5454.9
	(2469.6,2696.1)	(1147.6,1252.1)	(1598.1,1740.4)	(2499.8,2748.9)	(5060.3,5849.4)
**Average Expenditure per capita** (BDT)					
*NCD*	3060.7	1010.6	1583.1	2454.9	5908.4
	(2884.4,3237.0)	(987.9,1033.3)	(1557.5,1608.6)	(2418.5,2491.2)	(5508.1,6308.8)
*Non-NCD*	2414.8	1033.3	1579.5	2439.1	5170.0
	(2332.0,2497.6)	(1019.5,1047.0)	(1565.7,1593.4)	(2418.4,2459.8)	(4912.0,5427.9)
**Average Household Size** (No. of people)					
*NCD*	4.9	5.4	5.1	4.8	4.5
	(4.8,4.9)	(5.1,5.6)	(4.9,5.3)	(4.6,4.9)	(4.4,4.7)
*Non-NCD*	4.4	5.0	4.6	4.2	3.9
	(4.3,4.4)	(4.9,5.1)	(4.5,4.7)	(4.1,4.3)	(3.8,4.0)
**Presence of Elderly** (Prop.)					
*NCD*	0.362	0.380	0.348	0.395	0.326
	(0.341,0.383)	(0.320,0.440)	(0.308,0.389)	(0.361,0.430)	(0.287,0.366)
*Non-NCD*	0.210	0.199	0.216	0.201	0.227
	(0.200,0.220)	(0.177,0.222)	(0.199,0.233)	(0.185,0.217)	(0.204,0.251)
No. of Sample Households	12,214	1,755	3,796	4,175	2,506

^a^ 95% confidence interval in parentheses. *** p<0.01, ** p<0.05, * p<0.1.

^b^ NCD household status is defined as at least one household member reporting one or more of the following NCDs–heart disease, hypertension, diabetes, kidney diseases, asthma, cancer.

^c^ Household income categories are defined by household’s consumption per capita relative to the stratum specific consumption poverty line, as follows: very poor (<80% of the poverty line), marginally poor (> = 80% and <120% of the poverty line), marginally well-off (> = 120% and <200% of the poverty line), and well-off (> = 200% of the poverty line).

^d^ Arithmetic mean. Since households are already categorized in groups by their respective positions in the expenditure per capita distribution, we prefer to report the arithmetic mean for the group instead of median for the group.

We first evaluated the relationship between NCD diagnosis and household expenditure by obtaining the unadjusted differences in expenditure shares across NCD and non-NCD households. HIES 2010 survey weights are used to obtain unadjusted differences across household groups. Next, we estimated adjusted differences using a system of Engel curves in a Quadratic Almost Ideal Demand System (QUAIDS) framework developed by Banks, et. al., (1997) [20], as follows:

$$w_{ik}=\beta_{0}+\beta_{1}{NCD}_{i}+\beta_{2}\ln Y_{i}+\beta_{3}{\left(\ln Y_{i}\right)}^{2}+X_{i}\beta_{4}+Strata_{i}\Gamma +\varepsilon_{ik}$$ (1)

where *w*_*ik*_ denotes the expenditure share of the *k*^*th*^ commodity of household *i*, and Eq 1 was applied to 10 expenditure categories using seemingly unrelated regression (SUR) and feasible generalized least squares (FGLS) framework with the miscellaneous expenditure category omitted from the system to meet the summation restriction. *NCD*_*i*_ is a binary variable that takes the value 1 if NCDs are present in household *i*, and 0 otherwise. *Y*_*i*_ is household *i*'s monthly consumption expenditure, and ***X***_***i***_ is a vector of household demographic and socioeconomic characteristics including presence of other diseases or injuries in the household, household size, presence of children under age 5, presence of children aged 5 to 14, presence of elderly members (age 60+), proportion of males among household adults, household head’s gender, household head’s education level, household head’s main occupation, household’s major source of income, and household religion. Following Banks, et. al., (1997), the household expenditure terms ln*Y*_*i*_ and (ln*Y*_*i*_)^2^ were instrumented with household income, ln*M*_*i*_ and (ln*M*_*i*_)^2^, respectively [20]. ***Strata***_***i***_ represents stratum fixed effect and *ε*_*ik*_ is the idiosyncratic error term. In the cross section data, we do not observe price differences over time. Therefore, to estimate Engel curves, we assume that prices of respective commodities are same for households within the strata, and stratum fixed effects control for the differences in commodity prices across strata. This assumption is consistent with the official stratum-specific poverty line estimates in Bangladesh that consider differential cost of living across strata. Stratum fixed effects also controls for geographic and regional differences in access to healthcare services and treatment costs. The coefficient β_*1*_ describes the adjusted difference in household consumption shares between non-NCD and NCD households.

Finally, we analyzed differences in food expenditure shares across NCD and non-NCD households using SUR as follows:

$$y_{ij}=\alpha_{0}+\alpha_{1}{NCD}_{i}+\alpha_{2}\ln M_{i}+X_{i}\alpha_{3}+Strata_{i}\Gamma +\xi_{ij}$$ (2)

where *y*_*ij*_ denotes the food expenditure share of *j*^*th*^ food category of household *i*, and the remaining indicators are those described in Eq 1. The coefficient *α*_*1*_ describes the adjusted difference in food consumption shares between non-NCD and NCD households. Eqs 1 and 2 are separately estimated for the full sample, and for each of the sub-samples of household income category.

## Results

### NCD-associated differences for household consumption expenditure categories

Unadjusted differences between NCD and non-NCD households for 11 expenditure categories are shown in Table 2 for the full sample, and in Table 3 by household income category. The medical expenditure share for NCD households was 59% (1.78 percentage points (pp)) higher on average than that for non-NCD households. This relative difference was higher for the well-off households (72.5%), and lower (50.4%) for the very poor households. The food expenditure share, on the other hand, was 6% (3.49 pp) lower for NCD households than non-NCD households. NCD households were estimated to have higher expenditure shares for housing, education, and entertainment, and lower expenditure shares for clothing, lifestyle and hygiene, and energy.

**Table 2:** Unadjusted differences in expenditure categories between NCD and non-NCD households.

	Non-NCD Avg. (%)^a^	Difference (% points)^b^	Relative Diff. (%)
Medical	3.019	1.781***	58.988
	(2.872,3.166)	(1.430,2.132)	
Food	57.28	-3.493***	-6.099
	(56.684,57.876)	(-4.244,-2.742)	
Tobacco	2.868	-0.021	-0.725
	(2.704,3.031)	(-0.191,0.150)	
Clothing	5.729	-0.272***	-4.747
	(5.568,5.890)	(-0.441,-0.103)	
Housing	8.317	0.450*	5.415
	(7.905,8.729)	(-0.053,0.954)	
Education	3.739	0.996***	26.655
	(3.525,3.952)	(0.670,1.323)	
Lifestyle & Hygiene	3.129	-0.163***	-5.221
	(3.070,3.188)	(-0.246,-0.080)	
Energy	7.022	-0.623***	-8.874
	(6.851,7.194)	(-0.892,-0.354)	
Transportation & Communications	4.909	0.088	1.796
	(4.735,5.083)	(-0.183,0.359)	
Entertain	0.577	0.148***	25.603
	(0.527,0.628)	(0.066,0.229)	
Misc.	3.411	1.109***	32.502
	(3.203,3.619)	(0.723,1.494)	

^a^ 95% confidence interval in parentheses.

*** p<0.01

** p<0.05

* p<0.1

^b^ NCD household status is defined as at least one household member reporting one or more of the following NCDs–heart disease, hypertension, diabetes, kidney diseases, asthma, cancer.

**Table 3:** Unadjusted differences in expenditure categories between NCD and non-NCD households, by household type.

	Very Poor		Marginally Poor		Marginally well-off		Well-off	
	Non-NCD ^a^	Diff.	Non-NCD	Diff.	Non-NCD	Diff.	Non-NCD	Diff.
Medical	2.587	1.303***	2.742	1.122***	3.242	1.532***	3.487	2.529***
	(2.373,2.801)	(0.782,1.823)	(2.569,2.916)	(0.672,1.572)	(3.038,3.445)	(1.092,1.971)	(3.162,3.811)	(1.597,3.461)
Food	64.544	-1.000	61.526	-1.447***	56.510	-1.138**	44.341	-1.784**
	(63.834,65.255)	(-2.298,0.299)	(60.996,62.056)	(-2.374,-0.521)	(55.844,57.175)	(-2.002,-0.273)	(43.022,45.660)	(-3.373,-0.194)
Tobacco	3.015	0.409*	3.082	0.208	3.011	0.063	2.046	-0.097
	(2.802,3.228)	(-0.056,0.874)	(2.877,3.288)	(-0.084,0.499)	(2.787,3.234)	(-0.229,0.354)	(1.804,2.288)	(-0.357,0.163)
Clothing	6.196	-0.194	6.08	0.077	5.562	-0.027	4.979	-0.457***
	(5.903,6.489)	(-0.600,0.212)	(5.858,6.302)	(-0.237,0.390)	(5.387,5.736)	(-0.242,0.188)	(4.779,5.180)	(-0.715,-0.199)
Housing	4.992	-0.234	6.471	-0.388	8.343	-0.769**	14.696	-0.539
	(4.606,5.378)	(-0.864,0.395)	(6.062,6.880)	(-0.921,0.145)	(7.880,8.806)	(-1.364,-0.173)	(13.439,15.954)	(-1.994,0.915)
Education	1.993	0.229	2.635	0.510***	4.200	-0.031	6.461	1.338***
	(1.799,2.187)	(-0.255,0.712)	(2.442,2.828)	(0.145,0.875)	(3.921,4.480)	(-0.473,0.411)	(5.696,7.225)	(0.466,2.209)
Lifestyle & Hygiene	3.56	-0.104	3.296	-0.013	3.068	-0.056	2.554	-0.112
	(3.449,3.672)	(-0.319,0.112)	(3.223,3.369)	(-0.129,0.104)	(2.993,3.143)	(-0.171,0.059)	(2.451,2.658)	(-0.287,0.063)
Energy	8.638	-0.458*	7.694	-0.002	6.690	-0.285*	4.984	-0.407***
	(8.288,8.988)	(-0.980,0.063)	(7.479,7.909)	(-0.644,0.640)	(6.467,6.913)	(-0.574,0.004)	(4.779,5.189)	(-0.689,-0.126)
Transportation &	3.332	-0.034	4.337	-0.359**	5.319	-0.156	6.571	-0.248
Communications	(3.073,3.590)	(-0.543,0.475)	(4.140,4.533)	(-0.702,-0.016)	(5.080,5.559)	(-0.549,0.236)	(6.039,7.102)	(-0.970,0.474)
Entertainment	0.221	0.07	0.427	0.032	0.653	0.076	1.027	0.091
	(0.168,0.273)	(-0.061,0.200)	(0.369,0.485)	(-0.097,0.160)	(0.587,0.720)	(-0.068,0.221)	(0.893,1.161)	(-0.113,0.295)
Misc.	0.922	0.014	1.709	0.26	3.403	0.791***	8.854	-0.314
	(0.778,1.066)	(-0.420,0.449)	(1.558,1.861)	(-0.063,0.584)	(3.163,3.642)	(0.315,1.267)	(8.092,9.616)	(-1.450,0.822)

^a^ Notes: 95% confidence interval in parentheses.

*** p<0.01

** p<0.05

* p<0.1

NCD households were estimated to have lower expenditure on food across economic groups. Though the difference was not statistically significant for the very poor households, it ranged from 1.12 to 2.53 pp for the other sub-groups. The average tobacco expenditure share of the very poor NCD households was 13.6% (0.41 pp) higher than non-NCD households in the same income group. The clothing expenditure share was lower for NCD households in the well-off group only, and the housing expenditure share was lower for NCD households in the marginally well-off group only. The energy share of NCD households was lower for most household groups except for the marginally poor.

Adjusted differences from the regression analysis are reported in Table 4. The medical expenditure share was higher for NCD than non-NCD households in both the full sample and all sub-samples. The food expenditure share was lower for NCD households in the marginally poor group. The tobacco expenditure share was higher for NCD households in the full sample as well as for the well-off and marginally poor groups. The clothing expenditure share was lower for NCD households than non-NCD households across all groups, and the housing share was lower for all groups except the marginally poor. Adjusted differences in lifestyle and hygiene and energy were not statistically significant.

**Table 4:** Adjusted differences in expenditure categories between NCD and non-NCD households.

	All ^a^	Very Poor	Marginally Poor	Marginally Well-off	Well-off
Medical	1.519***	1.164***	1.066***	1.423***	2.366***
	(1.308, 1.730)	(0.749, 1.579)	(0.768, 1.364)	(1.077, 1.769)	(1.731, 3.001)
Food	-0.283	0.016	-0.697*	-0.518	0.514
	(-0.744, 0.178)	(-1.245, 1.277)	(-1.467, 0.074)	(-1.307, 0.271)	(-0.605, 1.632)
Tobacco	0.229***	0.319	0.262*	0.156	0.297**
	(0.086, 0.372)	(-0.103, 0.741)	(-0.010, 0.534)	(-0.097, 0.408)	(0.027, 0.567)
Clothing	-0.324***	-0.505**	-0.293**	-0.147	-0.390***
	(-0.445, -0.202)	(-0.944, -0.067)	(-0.537, -0.049)	(-0.337, 0.044)	(-0.627, -0.153)
Housing	-0.827***	-0.500*	-0.214	-0.906***	-1.732***
	(-1.177, -0.478)	(-1.065, 0.066)	(-0.658, 0.231)	(-1.433, -0.378)	(-2.901, -0.563)
Education	0.176	0.064	0.042	-0.158	0.665*
	(-0.068, 0.420)	(-0.351, 0.478)	(-0.277, 0.360)	(-0.569, 0.253)	(-0.070, 1.400)
Lifestyle and	-0.021	-0.053	-0.003	-0.002	-0.046
Hygiene	(-0.084, 0.042)	(-0.242, 0.136)	(-0.109, 0.102)	(-0.110, 0.105)	(-0.188, 0.095)
Energy	0.028	-0.274	0.149	-0.036	0.101
	(-0.098, 0.154)	(-0.692, 0.145)	(-0.103, 0.400)	(-0.249, 0.177)	(-0.100, 0.302)
Transportation &	-0.259**	-0.099	-0.313**	-0.189	-0.219
Communications	(-0.470, -0.047)	(-0.551, 0.354)	(-0.612, -0.015)	(-0.542, 0.165)	(-0.836, 0.397)
Entertainment	0.042	0.148**	0.008	0.03	0.057
	(-0.034, 0.118)	(0.009, 0.287)	(-0.097, 0.113)	(-0.095, 0.156)	(-0.172, 0.286)
No. of Obs.	12,214	1,755	3,796	4,157	2,506

^a^ 95% confidence interval in parentheses.

*** p<0.01

** p<0.05

* p<0.1

### NCD-associated differences for food expenditure sub-categories

Unadjusted differences between NCD and non-NCD households in food consumption shares are presented in Table 5. NCD households had lower food expenditures shares of cereal, oil, vegetables, and miscellaneous foods than non-NCD households, and higher shares of meat, milk, sugar, fruits, and beverages. Sugar, milk, and fruit expenditure shares were higher for NCD than non-NCD households by 25.5%, 22.9% and 26.3%, respectively. Oil and vegetable shares both are around 5% lower than those of non-NCD households. Table 6 reports the unadjusted differences in food expenditure shares by household sub-groups. The cereal share for NCD households was lower for the well-off group and higher for the marginally poor group. Positive differences between NCD and non-NCD households in sugar, milk, and fruit expenditure were statistically significant for the well-off and marginally well-off groups only. Negative differences in oil and vegetables were statistically significant for all groups except for the very poor. Lower shares of pulses (legumes) were found for the marginally well-off and marginally poor groups.

**Table 5:** Unadjusted differences in food expenditure categories between NCD & non-NCD households.

	Non-NCD Avg. (%)^a^	Difference (% Points)	Relative Diff. (%)
Cereal	42.878	-2.238***	-5.219
	(42.242,43.514)	(-3.074,-1.402)	
Pulses	2.669	-0.112*	-4.213
	(2.561,2.777)	(-0.225,0.000)	
Fish	13.674	0.142	1.040
	(13.349,14.000)	(-0.244,0.529)	
Egg	1.661	0.048	2.897
	(1.589,1.734)	(-0.052,0.148)	
Meat	6.672	1.193***	17.883
	(6.171,7.173)	(0.670,1.716)	
Milk	2.443	0.558***	22.853
	(2.296,2.590)	(0.366,0.751)	
Sugar	1.68	0.428***	25.499
	(1.567,1.793)	(0.286,0.571)	
Oil	4.992	-0.279***	-5.593
	(4.883,5.101)	(-0.383,-0.175)	
Fruit	3.287	0.865***	26.321
	(2.971,3.602)	(0.546,1.185)	
Vegetables	12.401	-0.667***	-5.379
	(12.180,12.622)	(-0.884,-0.450)	
Beverages	2.089	0.216***	10.322
	(1.957,2.221)	(0.078,0.353)	
Misc.	5.554	-0.154**	-2.776
	(5.434,5.674)	(-0.291,-0.018)	

^a^ 95% confidence interval in parentheses.

*** p<0.01

** p<0.05

* p<0.1

**Table 6:** Unadjusted differences in food expenditure categories between NCD and non-NCD households, by household type.

	Very Poor		Marginally Poor		Marginally well-off		Well-off	
	Non-NCD ^a^	Diff.	Non-NCD	Diff.	Non-NCD	Diff.	Non-NCD	Diff.
Cereal	54.481	0.387	47.299	1.181**	39.576	0.107	30.802	-1.174**
	(53.596,55.366)	(-1.197,1.972)	(46.636,47.963)	(0.014,2.348)	(38.979,40.173)	(-0.759,0.972)	(29.751,31.854)	(-2.304,-0.044)
Pulses/	2.37	-0.094	2.602	-0.239***	2.789	-0.197**	2.822	-0.033
Legumes	(2.186,2.554)	(-0.390,0.202)	(2.467,2.737)	(-0.417,-0.061)	(2.649,2.928)	(-0.362,-0.033)	(2.649,2.995)	(-0.233,0.167)
Fish	10.651	-0.303	12.702	-0.434	14.712	-0.553**	16.128	-0.098
	(10.123,11.178)	(-1.198,0.593)	(12.295,13.108)	(-1.056,0.187)	(14.293,15.131)	(-1.105,-0.001)	(15.509,16.747)	(-0.758,0.562)
Egg	1.12	-0.172*	1.484	-0.075	1.800	0.018	2.200	-0.083
	(1.012,1.227)	(-0.355,0.011)	(1.400,1.568)	(-0.236,0.086)	(1.702,1.898)	(-0.115,0.151)	(2.007,2.393)	(-0.333,0.168)
Meat	2.568	-0.111	4.612	-0.077	7.693	0.056	12.178	0.801
	(2.220,2.915)	(-0.793,0.572)	(4.190,5.035)	(-0.789,0.635)	(7.064,8.322)	(-0.636,0.747)	(11.076,13.279)	(-0.305,1.907)
Milk	0.87	0.03	1.831	0.023	2.878	0.386**	4.127	0.337*
	(0.731,1.009)	(-0.251,0.310)	(1.638,2.024)	(-0.296,0.341)	(2.671,3.084)	(0.078,0.694)	(3.841,4.412)	(-0.056,0.730)
Sugar	0.929	0.159	1.412	0.108	1.828	0.264**	2.558	0.473***
	(0.767,1.091)	(-0.074,0.392)	(1.280,1.543)	(-0.067,0.282)	(1.691,1.964)	(0.034,0.495)	(2.373,2.742)	(0.198,0.747)
Oil	5.172	-0.126	5.05	-0.267***	5.003	-0.255***	4.702	-0.216**
	(5.005,5.339)	(-0.417,0.166)	(4.919,5.182)	(-0.436,-0.098)	(4.880,5.126)	(-0.402,-0.108)	(4.540,4.864)	(-0.394,-0.038)
Fruit	1.634	0.124	2.478	0.251	3.580	0.517**	5.700	0.674***
	(1.313,1.955)	(-0.304,0.551)	(2.175,2.780)	(-0.163,0.665)	(3.221,3.938)	(0.105,0.930)	(5.089,6.310)	(0.186,1.163)
Vegetables	13.405	0.017	13.148	-0.485**	12.136	-0.294*	10.622	-0.473***
	(13.057,13.754)	(-0.527,0.561)	(12.863,13.433)	(-0.851,-0.118)	(11.868,12.403)	(-0.597,0.009)	(10.305,10.939)	(-0.780,-0.166)
Beverages	1.329	0.074	1.787	0.151	2.386	0.115	2.744	-0.033
	(1.169,1.489)	(-0.245,0.393)	(1.645,1.928)	(-0.058,0.360)	(2.205,2.567)	(-0.102,0.332)	(2.521,2.966)	(-0.283,0.216)
Misc.	5.471	0.015	5.594	-0.137	5.621	-0.163*	5.418	-0.175
	(5.263,5.680)	(-0.423,0.453)	(5.429,5.759)	(-0.384,0.110)	(5.477,5.765)	(-0.355,0.029)	(5.247,5.589)	(-0.424,0.073)

^a^ 95% confidence interval in parentheses.

*** p<0.01

** p<0.05

* p<0.1

Adjusted differences in food expenditure shares are reported in Table 7. Overall, NCD status was positively associated with expenditure on meat, milk, sugar, fruit, beverages and negatively associated with expenditure on pulses (legumes), cereal, fish, oil, and vegetables. Differences between NCD and non-NCD households in cereal expenditure were negative for the well-off sub-group and positive for the marginally poor sub-group. Negative differences in pulse (legume) share and oil share were statistically significant for the marginally well-off and marginally poor groups; and negative differences in fish share were statistically significant for the marginally poor group. NCD status was associated with higher sugar expenditure in the well-off and marginally well-off household groups.

**Table 7:** Adjusted differences in food expenditure categories between NCD and non-NCD households.

	All ^a^	Very Poor	Marginally Poor	Marginally Well-off	Well-off
Cereal	-0.883***	0.474	1.123***	-0.435	-0.903**
	(-1.296, -0.469)	(-0.649, 1.598)	(0.379, 1.867)	(-1.057, 0.187)	(-1.607, -0.199)
Pulses/	-0.128***	-0.064	-0.277***	-0.122*	-0.015
Legumes	(-0.210, -0.046)	(-0.332, 0.205)	(-0.438, -0.116)	(-0.256, 0.012)	(-0.168, 0.137)
Fish	-0.242*	-0.293	-0.458*	-0.225	-0.365
	(-0.502, 0.018)	(-1.036, 0.450)	(-0.937, 0.021)	(-0.659, 0.210)	(-0.896, 0.167)
Egg	0.056	-0.06	0.01	0.102	0.027
	(-0.025, 0.136)	(-0.266, 0.146)	(-0.118, 0.138)	(-0.021, 0.226)	(-0.194, 0.248)
Meat	0.566***	0.06	-0.207	-0.067	0.638
	(0.223, 0.909)	(-0.584, 0.703)	(-0.747, 0.334)	(-0.647, 0.513)	(-0.181, 1.456)
Milk	0.259***	0.011	0.025	0.252*	0.078
	(0.098, 0.420)	(-0.286, 0.308)	(-0.271, 0.320)	(-0.039, 0.543)	(-0.268, 0.425)
Sugar	0.282***	0.007	0.126	0.195**	0.359***
	(0.192, 0.372)	(-0.204, 0.218)	(-0.036, 0.288)	(0.044, 0.347)	(0.159, 0.558)
Oil	-0.168***	-0.113	-0.197***	-0.122**	-0.053
	(-0.243, -0.093)	(-0.355, 0.130)	(-0.346, -0.048)	(-0.239, -0.005)	(-0.194, 0.088)
Fruit	0.379***	0.021	0.006	0.301*	0.357
	(0.198, 0.560)	(-0.366, 0.408)	(-0.298, 0.310)	(-0.004, 0.607)	(-0.075, 0.789)
Vegetables	-0.194**	0.079	-0.104	0.07	-0.144
	(-0.347, -0.041)	(-0.383, 0.540)	(-0.411, 0.203)	(-0.174, 0.314)	(-0.404, 0.115)
Beverages	0.137**	-0.143	0.073	0.148	0.11
	(0.029, 0.246)	(-0.432, 0.145)	(-0.121, 0.268)	(-0.046, 0.342)	(-0.108, 0.328)
No. of Obs.	12,214	1,755	3,796	4,157	2,506

^a^ 95% confidence interval in parentheses.

*** p<0.01

** p<0.05

* p<0.1

## Discussion

Consumption displacement is one mechanism through which NCDs can affect the economic wellbeing of households. In Bangladesh, where health insurance coverage is limited, NCD-afflicted households may be vulnerable to added out-of-pocket medical expenditures, and may compensate for these by reducing consumption of other necessities. In this study, we use household-level data from Bangladesh to explore the association between presence of NCDs in the household and household resource allocation across broad expenditure categories (medical, food, housing, etc.) and across food sub-categories (sugar, meat, vegetables, etc.). Unadjusted and regression-adjusted expenditure differences between NCD and non-NCD households were estimated for the full sample and for four household sub-groups defined by economic status–very poor, marginally poor, marginally well-off and well-off.

NCD households were found to have larger medical expenditure shares than non-NCD households across all household groups, potentially displacing the consumption of other commodities. On average, NCD-afflicted households had lower expenditure on food, clothing, hygiene, and energy. Regression-adjusted results indicated that NCD status was associated with lower household spending on clothing and housing in all groups, and with lower food spending among the marginally poor, highlighting the disproportionate burden an NCD diagnosis might impose on households living near the poverty line. These circumstances can contribute to perpetuating the poverty cycle in Bangladesh by further impairing productivity and distorting the distribution of resources within the household.

One limitation of our study is that the analysis shows how consumption displacement and NCD status are associated, but cannot identify the causal channels through which NCDs could impact household consumption. NCDs could be associated with spending on unhealthy items, other comorbidities and productivity loss or loss of income, which may affect consumption decisions. NCD treatment could also alter or adjust food consumption and cause lifestyle changes. We cannot separately estimate the specific impacts of these factors on consumption displacement. These aspects require intertemporal analyses by observing households pre and post NCD affliction, which given the cross-section nature of our data are beyond the scope of this paper. This study entails analysis of the contemporaneous association between the presence of NCDs and household consumption patterns.

As expected, the consumption share of tobacco, a primary NCD risk factor, was greater for NCD households. Tobacco expenditure might contribute to the consumption displacement of essential goods not only directly but also through added medical expenditures from tobacco-related NCDs. NCD-afflicted households were found to allocate less on staples like fish, cereal, pulses, oil, and vegetables, and more on higher-calorie foods like sugar, meat, and beverages. After rice (a main type of cereal), fish and vegetables are the leading categories in the food consumption basket in Bangladesh. Lower fish and vegetable consumption among NCD-afflicted households might have health and economic effects that persist across generations by affecting the nutritional needs of children in the household. The nutritional allocations of NCD households relative to non-NCD households may be relevant for policy-makers concerned with dietary behavior and chronic disease in Bangladesh.

We observe that relatively well-off households are likely to report more cases of NCDs, compared to poorer households. This gradient might partially reflect under-diagnosis and lack of awareness of NCDs, which may occur disproportionately in poorer households given limited access to health care services. To address this possibility, we evaluated the role of NCD status in household resource allocation within four economic subgroups. The subgroup analysis could inform policies for integrated health interventions and nutrition programs for the population that are most vulnerable to consumption displacement. Our findings indicate that within all economic subgroups, households with NCD incidence remain at higher risk of consumption displacement of essential commodities. The association between NCD status and reduced food consumption among the marginally poor suggests potential nutritional consequences for economically vulnerable households. The findings indicate a link between NCDs and the possibility of adverse economic effects on households in Bangladesh, and may inform further public health interventions for NCD prevention and control.
